# Revealing Genomic Insights of the Unexplored Porcine Pathogen Actinobacillus pleuropneumoniae Using Whole Genome Sequencing

**DOI:** 10.1128/spectrum.01185-22

**Published:** 2022-07-20

**Authors:** Judith Guitart-Matas, Narjol Gonzalez-Escalona, Meghan Maguire, Anna Vilaró, Jaime Martinez-Urtaza, Lorenzo Fraile, Lourdes Migura-Garcia

**Affiliations:** a Joint Research Unit IRTA-UAB in Animal Health, Animal Health Research Centre (CReSA), Campus of the Autonomous University of Barcelona (UAB), Bellaterra, Catalonia, Spain; b IRTA, Animal Health Program, Animal Health Research Centre (CReSA), Campus of the Autonomous University of Barcelona (UAB), Bellaterra, Catalonia, Spain; c OIE Collaborating Centre for the Research and Control of Emerging and Re-Emerging Swine Diseases in Europe (IRTA-CReSA), Bellaterra, Barcelona, Spain; d Food and Drug Administration, Silver Spring, Maryland, United States; e Pig Sanitation Group, Lleida, Spain; f Department of Genetics, Autonomous University of Barcelona, Bellaterra, Spain; g Department of Animal Science, ETSEA, University of Lleida, Lleida, Spain; Texas A&M University

**Keywords:** *Actinobacillus*, whole genome sequencing, swine

## Abstract

Actinobacillus pleuropneumoniae (APP) is the causative agent of pleuropneumonia in pigs, one of the most relevant bacterial respiratory diseases in the swine industry. To date, 19 serotypes have been described based on capsular polysaccharide typing with significant virulence dissimilarities. In this study, 16 APP isolates from Spanish origin were selected to perform antimicrobial susceptibility tests and comparative genomic analysis using whole genome sequencing (WGS). To obtain a more comprehensive worldwide molecular epidemiologic analyses, all APP whole genome assemblies available at the National Center for Biotechnology Information (NCBI) at the time of the study were also included. An in-house *in silico* PCR approach enabled the correct serotyping of unserotyped or incorrectly serotyped isolates and allowed for the discrimination between serotypes 9 and 11. A pangenome analysis identified the presence or absence of gene clusters to be serotype specific, as well as virulence profile analyses targeting the *apx* operons. Antimicrobial resistance genes were correlated to the presence of specific plasmids. Altogether, this study provides new insights into the genetic variability within APP serotypes, correlates phenotypic tests with bioinformatic analyses and manifests the benefits of populated databases for a better assessment of diversity and variability of relatively unknown pathogens. Overall, genomic comparative analysis enhances the understanding of transmission and epidemiological patterns of this species and suggests vertical transmission of the pathogen, including the resistance genes, within the Spanish integrated systems.

**IMPORTANCE** Pleuropneumonia is one of the most relevant respiratory infections in the swine industry. Despite Actinobacillus pleuropneumoniae (APP) being one of the most important pathogens in the pig production, this is the first comparative study including all available whole genome sequencing data from NCBI. Moreover, this study also includes 16 APP isolates of Spanish origin with known epidemiological relationships through vertical integrated systems. Genomic comparisons provided a deeper understanding of molecular and epidemiological knowledge between different APP serotypes. Furthermore, determination of resistance and toxin profiles allowed correlation with the presence of mobile genetic elements and specific serotype, respectively.

## INTRODUCTION

Actinobacillus pleuropneumoniae (APP) is the pathogen responsible of one of the most important bacterial respiratory diseases in the swine industry worldwide (1). Thus, it is listed among the 10 most important pathogens affecting pig production (2). In most countries, this disease is not under official control, and therefore, exact and updated information about its prevalence is difficult to know since reporting cases is not mandated by law. Animals infected with APP can present a range of clinical symptoms that vary from acute to chronic and even subclinical, but when outbreaks occur in field conditions, a sudden increase in mortality is usually observed (1). Overall, this disease causes large economic losses to the pig industry due to increased mortality rates, reduced growth rates, and cost of the control measures such as antibiotic treatments and vaccines.

This pathogen presents a huge variability worldwide. Currently, 19 serotypes have been described for this pathogen that differ significantly in virulence (1, 3). Different factors such as management practices (weaning age for example), time of the year, and production systems are also risk factors associated with the emergence of this pathogen in each particular farm or pig integration company (4). Interestingly, 80% of the swine industry in Spain is integrated, with a hierarchical pyramid structure. Generally, these pyramids integrate vertically the different levels of the production within the same system but separating physically the different phases of the rearing cycle. For example, one company holds the genetic selection of breeders (grandmothers) in a farm with high-health status that supplies the gilts for multiplication farms. When these gilts are approximately 6 months of age, they are moved to the different multiplication farms (mothers) where they will be mated to provide piglets. After weaning, piglets of approximately 30 kg are transported to finishing farms, where they remain until slaughter for human consumption. These vertically integrated systems may explain the circulation of pathogens and resistance traits from the top of the pyramid (breeders) to the bottom (piglets). Furthermore, one can hypothesize that knowledge of the epidemiology of the APP circulating in the production pyramid may help to implement preventive medicine programs for an efficient control of the disease in the whole production system. Moreover, the European legislation on veterinary medicinal products recommends using epidemiological information as a sound criterion to select the most suitable antimicrobial to be used in each clinical case (5). Thus, it has been recently proposed to determine the MIC for a battery of antimicrobials in one clinical case and use these epidemiological data in future clinical cases if the sow origin is the same (6). However, this approach has not been validated from a scientific point of view.

Alternatively, the characterization of the APP isolates circulating in these integrated systems is essential to apply control measures, since commercial vaccines are usually based on bacterins that provide limited cross-protection between serotypes (7). Currently, the most common typing scheme for APP is based on the antigenic properties of the capsule polysaccharides, with 19 serotypes described up to date (3, 8). Virulence mechanisms conferred by the repeat-in-toxin (RTX) family (toxins Apx I to IV) are involved in the development of disease, providing different cytotoxic effects depending on the toxins produced by each particular APP. Additionally, serotype-specific secretion of Apx toxins is commonly reported (9–11). Based on these typing methods, several studies have attempted to describe the epidemiology of the disease in different countries (12, 13). Recently, whole genome sequencing (WGS) has also been performed to unravel the genomic variability of APP, but mainly for serotype 8, and a scarce number of APP isolates have been sequenced over the last few years (14, 15). To our knowledge, there is a lack of studies applying WGS for epidemiological studies of this disease, and there are few APP DNA sequences published in public databases, especially of Spanish origin. Therefore, the first aim of this study is to apply comparative genomics to assess virulence, resistance, and phylogeny of all available isolate assemblies in public databases. Furthermore, Spanish APP lineages in related farms are epidemiologically analyzed to determine the transmission of this respiratory pathogen within these integrated systems. Finally, WGS has been used to demonstrate the suitability of the epidemiological approach to support prudent use of antimicrobials for this bacterium in swine medicine.

## RESULTS

### General genomic features of selected isolates.

**FIG 1:**
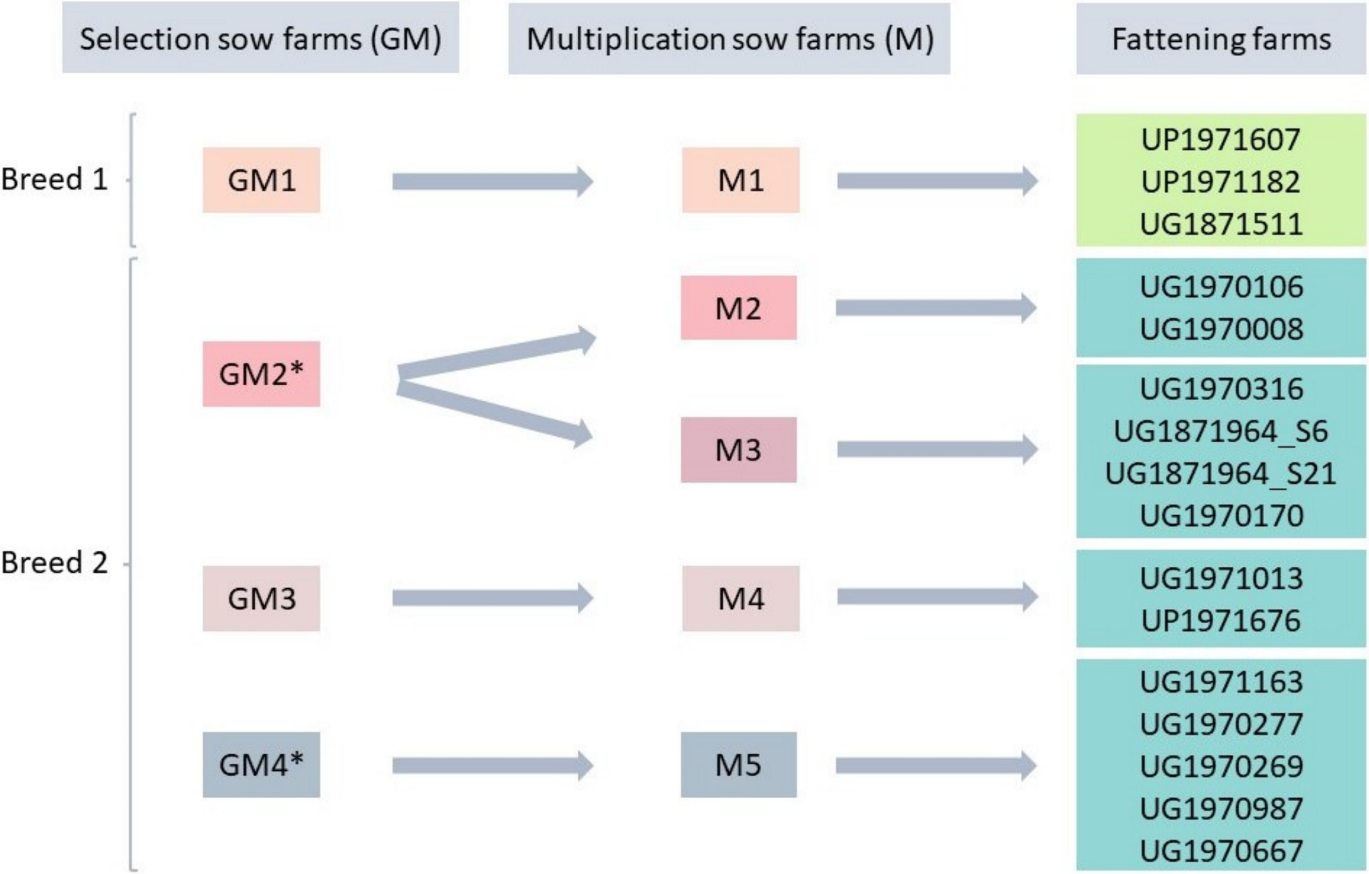
Epidemiological link between fattening, multiplication or mothers' farm (M), and selection or grandmothers' farm (GM) for the 16 APP isolates selected for whole genome sequencing analysis. GM2 and GM4 (*) farms shared the same origin of sows during foundation of both farms.

Whole genome sequencing data from the 16 APP isolates belonging to this study (Fig. 1) allowed the generation of genome assemblies with high quality. A total set of 31 additional APP assemblies available in NCBI at the time of the study (April 2021) was downloaded and checked for quality. Only one sample was discarded because its genome length was four times longer compared to the rest of the genomes. The final list of 46 isolates included in the analysis is detailed in Table 1.

**TABLE 1:** List of isolates selected for the analysis (*n* = 46) and metadata information including serotype, country of isolation, and year of isolation^*a*^

	This study							
ID	Serotype	Country of isolation	Yr of isolation	Selection farm (GM)	Multiplication farm (M)	Biosample	SRA accession	WGS accession
UP1971607	13	Spain	2019	1	1	SAMN23242902	SRR16969607	JAJMUP000000000
UP1971182	13	Spain	2019	1	1	SAMN23242903	SRR16969606	JAJMUO000000000
UG1871511	13	Spain	2018	1	1	SAMN23242904	SRR16969605	JAJMUN000000000
UG1970106	11	Spain	2019	2	2	SAMN23242905	SRR16969618	JAJMUM000000000
UG1970008	11	Spain	2019	2	2	SAMN23242897	SRR16969612	JAJMUU000000000
UG1970316	11	Spain	2019	2	3	SAMN23242906	SRR16969617	JAJMUL000000000
UG1871964_S21	11	Spain	2018	2	3	SAMN23242907	SRR16969616	JAJMUK000000000
UG1871964_S6	11	Spain	2018	2	3	SAMN23242898	SRR16969611	JAJMUT000000000
UG1970170	11	Spain	2019	2	3	SAMN23242901	SRR16969608	JAJMUQ000000000
UG1971013	11	Spain	2019	3	4	SAMN23242896	SRR16969619	JAJMUV000000000
UP1971676	11	Spain	2019	3	4	SAMN23242908	SRR16969615	JAJMUJ000000000
UG1971163	11	Spain	2019	4	5	SAMN23242909	SRR16969614	JAJMUI000000000
UG1970277	11	Spain	2019	4	5	SAMN23242900	SRR16969609	JAJMUR000000000
UG1970269	11	Spain	2019	4	5	SAMN23242899	SRR16969610	JAJMUS000000000
UG1970987	11	Spain	2019	4	5	SAMN23242910	SRR16969613	JAJMUH000000000
UG1970667	11	Spain	2019	4	5	SAMN23242895	SRR16969620	JAJMUW000000000
	**NCBI assembly database**							
**ID**	**Serotype**	**Country of isolation**	**Yr of isolation**	**Strain**	**GenBank accession**	**Reference**		
42650_C01	4	USA	1980	NCTC11384	LS483358.1	Kilian, M. et al. (16)		
42650_D01	6	Denmark	1971	NCTC11407		Pohl, S. et al. (17)		
56750_E01	2	Denmark	1973	NCTC10976	LR134515.1			
ASM1588v1	5		2007	L20	CP000569.1	Foote, S. et al. (18)		
ASM1668v1	3	China	2008	JL03	CP000687.1	Xu, Z. et al. (19)		
ASM16709v1	5	Argentina	2003	4074		Xu, Z. et al. (20)		
ASM1676271v1	1	China	2017	1140		No reference		
ASM1735746v1	5	China	2021	App6	CP026009.1	No reference		
ASM17849v2	1	Argentina	2018	4074	CP029003.1	Xu, Z. et al. (20)		
ASM17851v1	2		2010	S1536				
ASM17853v1	4	Australia	2010	M62				
ASM17855v1	6	Australia	2010	Femo				
ASM17857v1	9	Netherlands	2010	CVJ13261				
ASM17859v1	10		2010	D13039				
ASM17861v1	11	Netherlands	2010	56153				
ASM17863v1	12	Australia	2010	1096				
ASM17865v1	7	Hungary	2010	N273		Xu, Z. et al. (20)		
ASM17927v1	2	Australia	2010	4226		Zhan, B. et al. (21)		
ASM17929v1	6	Australia	2010	Femo				
ASM2040v1	7	Canada	2008	AP76	CP001091.1	Xu, Z. et al. (20)		
ASM29591v1	7	China	2012	S8		Li, G. et al. (22)		
ASM329038v1	1	Argentina	2018	S4074	CP030753.1	Dona, V. and Perreten, V. (23)		
ASM343140v1	1	South Korea	2017	16:00	CP022715.1	No reference		
ASM81744v1	8	Brazil	2011	1022		Pereira, M. et al. (24)		
ASM81746v1	8	Brazil	2007	460				
ASM81748v1	8	Brazil	2007	518				
ASM81751v1	8	Brazil	2009	780				
ASM81752v1	8	Brazil	2006	5651				
ASM81753v1	8	Brazil	2008	597		Pereira, M. et al. (24)		
MIDG2331	8	UK	2015	MIDG2331	LN908249.1	Bossé, J.T. et al. (25)		

a A total of 16 isolates derived from this study and metadata also includes both selection and multiplication origin farms. Separately, 30 isolates were selected from the NCBI database.

The genome of APP strain S4074 available at NCBI (GenBank accession number CP030753.1) with a genome size of 2,318,257 bp and GC content of 41.24%, was used as the representative and reference for that species for the analysis. The results obtained from the QUAST analysis of all the assemblies determined that the average genome size for this species was 2.3 Mbp and the GC content ranged from 41.16% to 41.20%.

### Serotyping using in-house *in silico* PCRs.

The serotype of four APP of the data set downloaded from NCBI was not available or was not included in the description. The assembly IDs of these isolates were: 42650_C01 (strain NCTC11384), 42650_D01 (strain NCTC11407), 57675_E01 (strain NCTC10976), and ASM1676271v1 (strain 1140). *In silico* PCR allowed the accurate serotyping of these isolates that were identified as serotypes 4, 6, 2, and 1, respectively. Moreover, two isolates were found to be incorrectly linked to a specific reference strain and corresponding serotype. The genome assemblies ASM16709v1 and ASM17865v1, linked to strains 4074 and N273, respectively, were correctly identified as serotypes 5 and 7. Separately, the alignment of the total 15 isolates belonging to serotypes 9 and 11 showed a base deletion at the end of the *cps* gene (*cpsF*) in all of them, except for the known serotype 9 isolate (strain CVJ13261). Moreover, the length of the PCR product for these isolates was 1,242 bp, confirming Spanish isolates from M2 to M5 as serotype 11.

### Phylogenetic reconstruction and *in silico* determination of virulence and antimicrobial resistance profiles.

**FIG 2:**
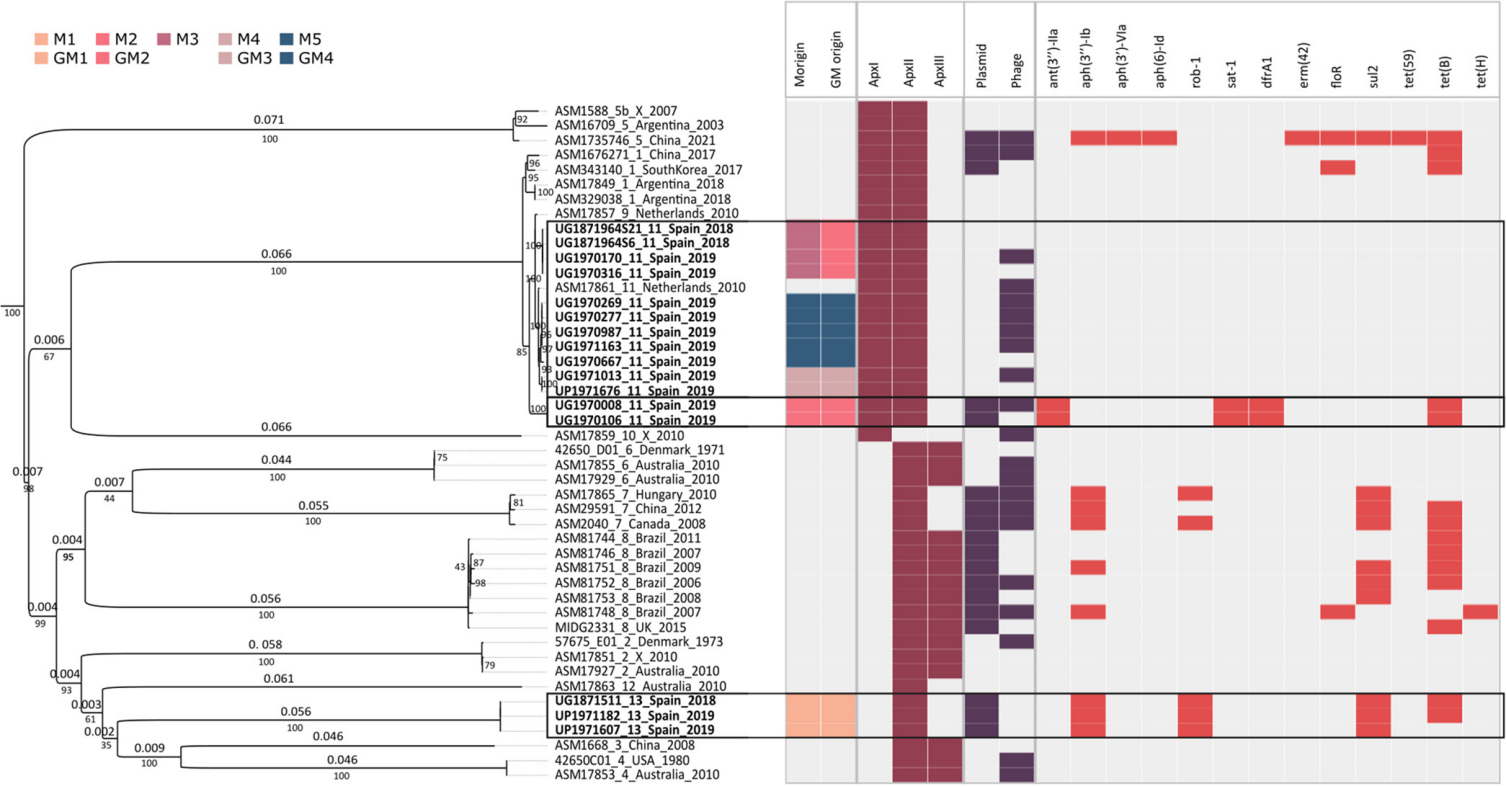
Virulence and resistance profile of the 46 APP isolates in a phylogenetic reconstruction. Columns from left to right indicate, multiplication farm (M), selection farm (GM), virulence profile including apxI, apxII and apxIII genes, plasmid and phage presence, and resistance profile. Phylogenetic reconstruction based on SNPs is represented on the left panel. Branch lengths and bootstrap support values are represented on the top and bottom of branches, respectively. Origin legend is specified on the top left of the figure. Serotype of each isolate is specified in the term before the country origin information of the isolate IDs.

To infer the phylogenetic relationships between the APP isolates, the core genome alignment based on SNPs was used. A total of 8,028 sites were used to construct the phylogenetic tree depicted in Fig. 2. Despite the low number of available isolates per serotype, distinct clades were clearly grouped by serotype. As previously reported, the virulent isolates of serotypes 1, 9, and 11 were aggregated in a common clade. Matrix counts of dissimilarity identified a maximum of 140 counts between isolates belonging to these serotypes. Longer distances were observed between this clade and the rest of serotypes, all of them above 2,000 counts of dissimilarity. The following serotype group with highest dissimilarity was serotype 5, with 183 maximum counts, followed by serotypes 7 and 8 with a maximum of 39 counts per cluster (Table S1).

Within Spanish isolates, breeds 1 and 2 were clearly split into two different clusters. Isolates collected from finishing farms supplied by M1 were considered identical. All isolates belonging to breed 2 were serotype 11 and were detected in farms supplied by M2 to M5. Another observation within this cluster was the presence of the Dutch isolate among Spanish isolates from multiplication farms M3 and M5 (Table S1). This geographical dispersion was also observed in other clusters, such as the relevant similarity between serotype 6 isolates from Australia and the Danish strain. This similarity also occurred within serotype 2, and within serotypes 7 and 4, from Canada and USA, respectively.

Recombination tools did not detect recombination accurately, as putative events were masked with regions with high density of base substitutions. This observation suggested that the different serotypes are remarkably diverse, and the data set should be split into closer related serotypes to appropriately assess recombination. Moreover, root-to-tip regression analyses suggested weak temporal signal, insufficient to proceed with evolutionary analyses.

**FIG 3:**
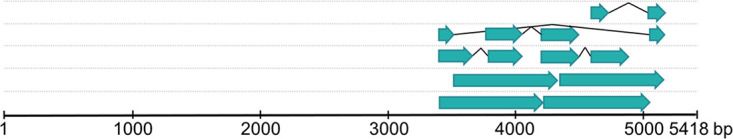
Repeat units detected by UGENE in nucleotide sequence of apxIV CDS from GenBank accession number AF021919.1. Each repeat unit is represented with a turquoise arrow and matches are linked with black lines. All repeat units share 97% of identity or higher.

The previously reported correlation between specific serotypes and the presence of Apx toxin genes was confirmed for *apxI*, *apxII*, and *apxIII* genes, as represented in Fig. 2. For these annotated genes, the identity was higher than 98% and the coverage was 100%. The *in silico* analysis for the presence of the *apxIV* gene, which codes for the ApxIV toxin, known to be secreted by all serotypes, produced inconsistent results regarding coverage percentages. Hence, as repeat units are known to lead to assembly errors, these features were further studied with the UGENE repeat analysis tool. Results showed that the *apxIV* gene contains many repeat regions that made its *de novo* assembly difficult. Fig. 3 depicts these regions in a 1,759 bp window with several repeat units from positions 3,388 to 5,146 of the *apxIV* gene.

In addition, analysis with the Virulence Factor DataBase (VFDB) identified only one virulence factor present in all 46 A. pleuropneumoniae isolates. This virulence factor is the phosphoheptose isomerase (gmhA/lpcA).

The *in silico* antimicrobial resistance profiles analysis for the 46 APP, detected using the CARD database, identified the presence of 13 resistance genes (Fig. 2). A total of 18 isolates harbored at least one gene conferring antimicrobial resistance to different antimicrobials correlated to the presence of plasmids (Table 2). Interestingly, a correlation was observed between isolates from serotypes 13 and 7, known to have the same Apx profile, and a similar resistance profile. Isolates from serotype 8 presented more variability, but all of them harbored at least one resistance gene. Two of the three serotype 5 isolates did not yield resistant genes, while the most recent isolate from 2021 (ASM1735746v1), carried a plasmid and harbored 8 resistance genes.

**TABLE 2:** Plasmids identified with PLSDB with a minimum identity of 0.98 and resistance genes known to be harbored in those plasmids^*a*^

Isolate ID	NCBI RefSeq	Length (bp)	GC (%)	Taxon	Plasmid name	Resistance genes
UG1871511_13_Spain_2018	NC_012215.1	4237	48.12	Pasteurella multocida	pB1005^*b*^	*sul2* *strA*
UP1971182_13_Spain_2019						
UP1971607_13_Spain_2019						
ASM343140_1_SouthKorea_2017	NZ_CP022716.1	7699	60.93	*APP*	unnamed1	*floR*
ASM1676271_1_China_2017	NZ_MT230378.1	1179	42.83	E. coli	pESBL87	*tet(B)*
UG1970008_11_Spain_2019	NC_013546.1	5486	40.09	*APP*	p11745	*tet(B)*
UG1970106_11_Spain_2019						
ASM1735746_5_China_2021	NZ_KX434882.1	4848	58.75	Klebsiella pneumoniae	pKP2442_7c331	*floR*
ASM2040_7_Canada_2008	NC_010942.1	5685	41.5	*APP*	APP7_A^*c*^	*rob-1*
ASM17865_7_Hungary_2010	NC_019176.1	4613	41.45	Haemophilus influenzae	pB1000^*c*^	*rob-1*
ASM29591_7_China_2012	NC_007098.1	3156	46.51	*APP*	pKMA2425	*sul2*
MIDG2331_8_UK_2015	NZ_MT230378.1	1179	42.83	E. coli	pESBL87	*tet(B)*
ASM81744_8_Brazil_2011	NZ_MH457196.1	5128	35.61	*APP*	p780	*tet(B)*
ASM81746_8_Brazil_2007						
ASM81748_8_Brazil_2009	NZ_KT355773.1	3937	52.78	*APP*	p518	*floR* *aph(3″)-Ib*
ASM81751_8_Brazil_2006	NZ_MH457196.1	5128	35.61	*APP*	p780	*tet(B)*
ASM81752_8_Brazil_2008	NC_009625.1	4065	45.41	*APP*	pARD3079	*sul2*
ASM81753_8_Brazil_2007						

a Only isolates identified with a plasmid are listed. Results from plasmidSPAdes were in agreement with PLSDB.

b Resistance region was 100% identical to the ABB7_B plasmid locus of APP.

c Alignment of both plasmid sequences shared 81% coverage with 99.8% of identity.

Regarding the Spanish isolates, sequencing data showing the presence of resistance genes agreed with the phenotypic results obtained by minimal inhibitory concentration (MIC). Three distinct phenotypes were identified (Table S2): i) resistance to amoxicillin, doxycycline, enrofloxacin, marbofloxacin, and oxytetracycline in all isolates of serotype 13 supplied by multiplication farm M1, harboring plasmid pB1005 associated to Pasteurella multocida (Table 2); ii) a pan-susceptible profile corresponding to 11 isolates of serotype 11; and iii) the remaining two isolates of serotype 11 (UG1970106 and UG1970008) that exhibited resistance to sulfamethoxazole/trimethoprim, doxycycline, and oxytetracycline. These two isolates were supplied by multiplication farm M2 where a tetracycline treatment was employed and harbored the plasmid p11745.

A total of 21 potential active prophages were detected among the 46 APP isolates with the Prophage Hunter tool (Table S3). The most common prophage identified was the *Mannheimia* phage (*n* = 18). The remaining three prophages identified were *Lactobacillus*, Streptococcus, and *Stenotrophomonas* phages. All of them had a score above 0.84.

Artemis visualization of genomic islands did not show a pathogenicity island pattern among isolates or serotypes. Between 20 and 32 genomic islands were predicted by the Alien Hunter software for each isolate’s genome and the lengths of these islands were also diverse (data not shown).

### Pangenome analysis.

Pangenome analysis with anvi’o identified a total of 3,200 gene clusters and 102,596 gene calls. The core and soft-core bins, including gene clusters shared by more than the 99% or between 95 to 99% of the isolates, respectively, contained 1,826 and 80 gene clusters, respectively. In addition, the shell bin, comprising gene clusters present in 15 to 95% of the isolates, contained 461 gene clusters, while the cloud bin, representing the gene clusters present in less than the 15% of the isolates, contained 833 gene clusters.

**FIG 4:**
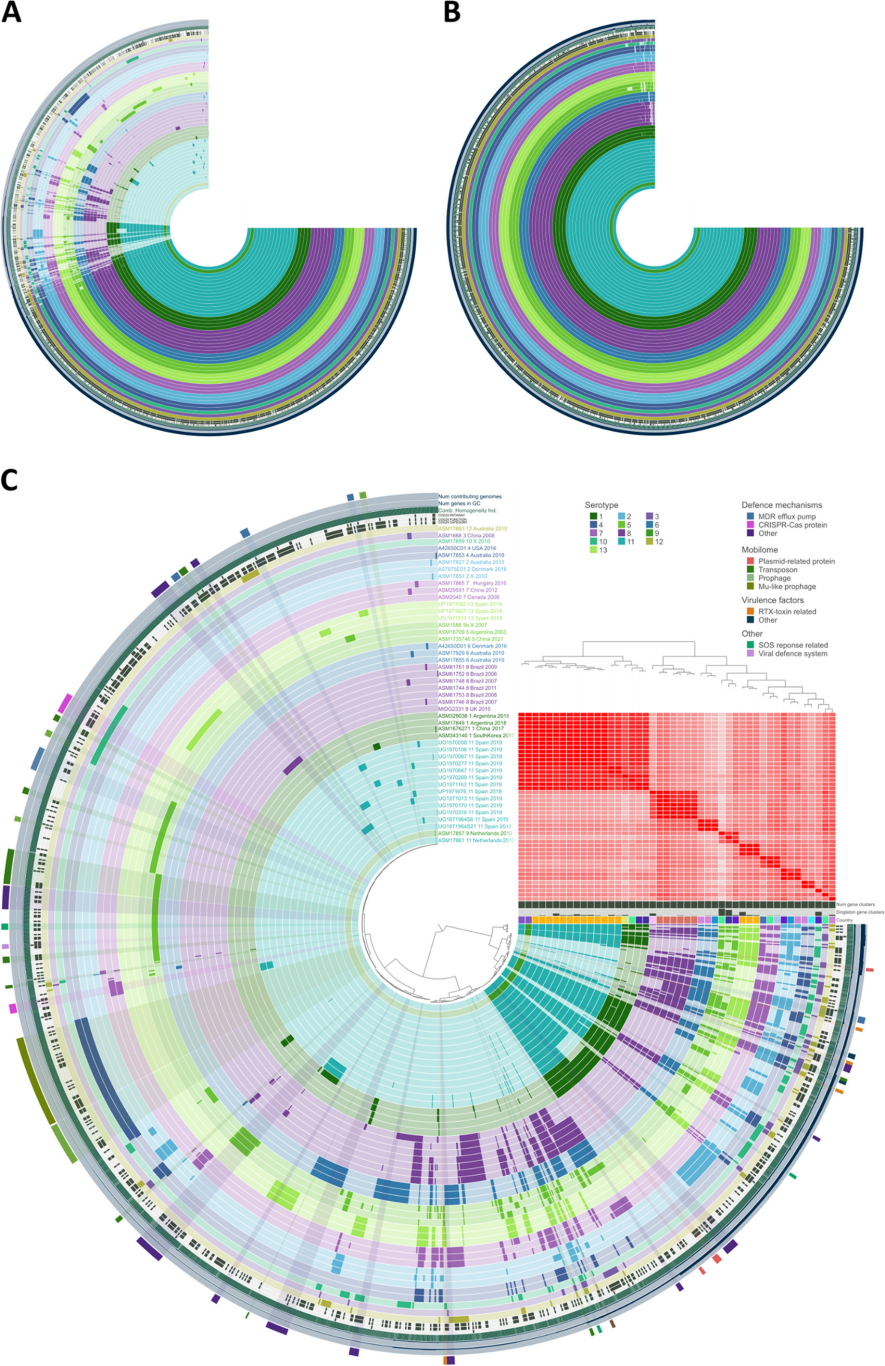
Pangenome analysis representation with anvi'o software. Isolates are colored by serotyped and ordered by presence/absence of gene clusters. (A) Full-pangenome representation including the core, soft-core, shell, and cloud bins. (B) Core representation, including the core and soft-core bins. (C) Accessory representation, including cloud and shell bins. Red and white matrix represents ANI values between isolates.

For proper identification of specific gene calls and clusters of orthologous gene (COG) functions, the full pangenome was split into two independent pangenomes: the core, including the gene clusters of the core and the soft-core, and the accessory, including the shell and the cloud gene clusters. Fig. 4A represents the full pangenome, while Fig. 4B only includes core and soft-core gene clusters. Shell and singleton gene clusters, representing the accessory, are shown in Fig. 4C.

Selected APP isolates were ordered by presence/absence of gene clusters and colored by serotype. As observed in Fig. 4C, a matrix representing ANI values correlates with distribution of presence/absence of gene clusters and serotype identification. Moreover, the previously observed clustering and cross-reactivity of serotypes 1, 9, and 11 is also detected in this matrix. This block is clearly clustered apart from the other serotypes, and includes, from right to left, the four isolates of serotype 1, the 13 isolates of this study identified as serotype 11, and the two isolates from The Netherlands, belonging to serotypes 9 and 11.

From the accessory bin, which included the gene clusters represented in less than 95% of the isolates, specific gene calls were identified, and COG functions putatively related to resistance and virulence were annotated. Within the accessory bin, the shell (gene clusters presented in 15% to 95% of the genomes) integrated several gene calls related to defense mechanisms and virulence factors, such as proteins VagC or RhuM, and proteins related to RTX toxins. Also, plasmid-related proteins were identified, such as proteins ParE, HigB, and VapI, transposon-related proteins, the SOS response repressor LexA, and multidrug resistance (MDR) efflux pumps.

The cloud (gene clusters presented in less than 15% of the genomes) included gene calls associated to defense mechanisms, transposon-related proteins, and MDR efflux pumps. Nevertheless, additional interesting features were identified in specific isolates’ genomes. The two isolates of serotype 2 harbored a large gene cluster of Mu-like prophage-encoded proteins and other prophage proteins were identified in assemblies ASM16709 (serotype 5) and ASM17859 (serotype 10). The later one also integrated CRISPR-Cas-related proteins, that were also identified in both isolates of serotype 7. In addition, a viral defense system was also annotated in a large gene cluster of strain App6 (GenBank accession number CP029003.1, assembly ASM1735746).

## DISCUSSION

Actinobacillus pleuropneumoniae (APP) causes one of the most economically relevant infectious diseases in swine production. Proper serotyping and identification of resistance and virulence profiles are essential to optimize preventive medicine programs based on antimicrobial and vaccine use. Furthermore, genomic comparisons among serotypes provide relevant molecular and epidemiological knowledge to better understand diversity and transmission patterns of this bacterial species across the swine production system. However, to date, this is the first study to include all the available sequencing data from NCBI for APP. Moreover, this comparative analysis includes the first WGS data of APP isolated from Spain.

Regarding the epidemiological link between Spanish farms, the phylogenetic tree clustered isolates from breed 1 and breed 2 in separate branches highlighting the presence of different lineages of APP circulating in Spanish farms. As observed, the three isolates from the three different farms supplied by GM1 and M1 during 2018 and 2019 were genetically related (0–1 nucleotide dissimilarity [Table S1]), suggesting that the pathogen is persisting and transmitted between the different production levels in a vertical way. Inversely, isolates from breed 2 that came from the GM2 did not cluster together in the phylogenetic tree. This observation is interesting as it was known that in the multiplication farm M2, as opposite to multiplication farm M3, animals were treated with oxytetracycline for a long period of time due to concomitant problems of leptospirosis (Fraile L, personal communication). The two isolates obtained from two different farms (UG1970008 and UG1970106) and supplied by M2 carried a plasmid and harbored four different resistance genes, including tetracycline resistance, hence, sustaining further the vertical transmission and supporting the evidence of resistance acquisition during or after antimicrobial treatment. On the contrary, isolates from breed 2 from selection farms GM3 and GM4 were phylogenetically related and clustered together in the tree (1–128 nucleotide dissimilarities [Table S1]) suggesting periodical introductions of the pathogen with no signals of adaptation to the different farm environments. Interestingly, the two isolates from The Netherlands (CVJ13261 and 56153) also appeared closely related to the Spanish isolates from breed 2 (40–114 nucleotide dissimilarities [Table S1]). This association may be explained by the movement of breeders across Europe, since The Netherlands is one of the largest exporters of live pigs globally, together with Denmark, Canada, and Germany (26). This same hypothesis may explain the association of specific isolates clustering together by country and serotype. For example, some Danish isolates appeared to be related to Australian isolates of serotypes 2 and 6, whereas the Canadian isolate of serotype 7 seems to be related to Chinese and Hungarian isolates of the same serotype. Regardless, the total number of isolates is extremely low to go further with these epidemiological links, but WGS paves the way to carry out wide epidemiological studies when plenty of sequences will be available.

APP is relatively understudied using WGS and it hampers the automation of the bioinformatic analysis pipeline. The absence of this pathogen in several databases, such as PointFinder to study point mutations, and the unavailability of the RTX toxin encoding genes in the reference virulence factor database (VFDB), made the downstream analyses from assembled genomes more complex and demanding (27, 28). Another challenge for the automation of virulence genes detection was the presence of tandem repeats in the *apxIV* toxin encoding gene. It is well recognized that these types of repeats lead to sequencing errors that make genome assembly and annotation difficult (29). Therefore, even after setting the parameters of identity at 90% and the coverage at 50%, we were unable to find the *apxIV* encoding gene in approximately 20% of the isolates, which did not correlate per serotype or per genotype. A more in-depth analysis found fractions of this gene located at the end or beginning of contigs and identified the *apxIV* encoding gene in all APP isolates. These observations associated the inability to detect the gene in some isolates to sequencing errors due to the presence of repetitive regions in the gene.

The implementation of *in silico* PCRs in this analysis allowed the serotyping of unserotyped isolates uploaded in NCBI, as well as the identification of incorrectly serotyped isolates. From these findings, no phylogenetic association was observed between serotypes 7 and 13 in disagreement with previously reported studies (20). *In silico* PCR demonstrated that strain N273 was incorrectly serotyped and belonged to serotype 7. This result was also supported by the phylogeny tree. Additionally, this technique enabled the proper discrimination between serotypes 9 and 11. These serotypes have always been detected with the same PCR primers, despite the fact that the conventional PCR cannot distinguish a single base deletion and has difficulties with less than 100 bp difference in a >1 kb product (3). The clustering of these two serotypes with serotype 1 in both pangenome reconstruction and phylogenetic tree corresponded with previously reported cross-reactivity between these three virulent serotypes (30, 31). Therefore, we have set the parameters *in silico* to accurately differentiate serotypes for a proper characterization of isolates.

The antimicrobial resistance *in silico* profiles did not show a clear serotype-specific correlation, as it has been already observed in other studies (32, 33). However, the presence of AMR genes was evidently linked to the presence of plasmids. Many of the resistance genes detected, including *aph(3″)-Ib*, *rob-1*, *floR,* and *sul2* were located in mobile genetic elements that would facilitate the transmission of the resistance. In fact, the reconstruction of these plasmids showed high homology with those described for pathogens of the *Pasteurellaceae* family, suggesting that transfer of genetic material between different genera of this family may occur easily. Similarly, the most common identified prophage from the Mannheimia haemolytica species is also a causative agent of a respiratory disease in bovine, and also a pathogen from the *Pasteurellaceae* family, like the genera *Actinobacillus*, *Pasteurella*, and Haemophilus. Interestingly, the two antimicrobial susceptibility patterns identified for the Spanish serotype 11 isolates correlated with the antimicrobial resistance *in silico* profiles: a clear pan-susceptible group and a group resistant to diaminopyrimidine and tetracycline antibiotics. However, for serotype 13 isolates, two *in silico* identified genes (*rob-1* and *sul2*) did not correspond with the phenotypic susceptible pattern observed in the sensitivity tests, suggesting the nonexpression and noninduction of these genes.

Overall, the genomic comparative analyses performed in this study make a significant contribution to the knowledge of the different serotypes of APP and corroborate toxin profile specificity per serotype. It also highlights the importance of databases being upgraded for automation of the bioinformatic analyses, especially for relatively unknown, albeit relevant, pathogens. Moreover, it increases understanding of phylogenetic-related serotypes and raises awareness of antimicrobial resistance genes and their potential to be transmitted through mobile genetic elements. Also, the epidemiological evidence observed clearly suggests a vertical transmission of these resistance traits within integrated systems, facilitating an epidemiological approach for early treatment of the disease in affected farms. Altogether, the study of the genetic variability between all available assemblies of APP at the time of the study demonstrated considerable differences within serotypes and provided information about prevalence of resistance among this community, strengthening the knowledge for proper surveillance and control of this pathogen.

## MATERIALS AND METHODS

### Selection of clinical samples, sequencing, assembly, and annotation.

Sixteen APP strains were isolated from deceased or diseased pigs showing acute clinical signs of respiratory tract infections. These animals were not exposed to any antimicrobial treatment for at least 15 days before sampling. Selection was based on the availability of the complete epidemiological link between 16 different fattening farms (origin of the isolates), multiplication or mothers’ farm (M), and grandmothers’ farm (GM) detailed in Fig. 1. Two breeders were identified from each selection farm and isolates were collected from 2018 onwards.

The DNA from the 16 selected APP isolates was extracted using the DNeasy Ultraclean Microbial Kit (Qiagen) according to the manufacturer’s instructions. Genomes were sequenced using the Illumina MiSeq platform (Illumina Inc., San Diego, CA) and with a MiSeq V3 kit using the 2 × 250 bp paired end chemistry.

Sequencing reads were identified for potential species contamination using Kraken v1.1.1 (34). The trimming step was performed using Trimmomatic v0.39 with a four-base sliding window to cut when the average Phred quality scores dropped below 15. Bases at the start or at the end of the reads with a quality below 3 were also removed and reads shorter than 36 bp were excluded (35).

Draft genomes were assembled *de novo* using the SPAdes assembler v3.14.1 including the module for preliminary read error correction based on Hamming graphs and Bayesian subclustering (36). K values and Phred scores were set to be automatically detected and the mismatch corrector tool BWA was also included. Quality evaluation of assemblies was performed using BUSCO v4.1.4 and QUAST v5.0.2 software (37, 38). Genomes were annotated using Prokka v1.14.6 (39).

### External data.

Additionally, all the assemblies available for APP (taxid:715) at the National Center for Biotechnology Information (NCBI) were downloaded (May 2021) and included for further analyses (40) (Table 1). Quality assessment of these assemblies was also performed with QUAST v5.0.2 and annotated with Prokka v1.14.6 (20, 30).

### *In silico* serotyping.

Unserotyped genome assemblies downloaded from NCBI were serotyped *in silico* using the Unipro UGENE *in silico* PCR tool (41). Already serotyped assemblies were also analyzed. Primer pairs used for specific detection of APP serotypes are described in Bossé, J.T., et al. (3). The number of maximum mismatches was set at 3 bp for both forward and reverse primers and the minimum 3′ perfect match was also set at 3 bp. Furthermore, all isolates identified as serotypes 9 or 11 were further analyzed for proper discrimination. This analysis included the 13 Spanish serotype 11 isolates and the APP strains CVJ13261 and 56153, belonging to serotypes 9 and 11, respectively. *In silico* PCR products were aligned using the UniPro UGENE alignment tool to detect the single base deletion in the final *cps* gene in serotype 11 reported by Bossé J.T., et al. (3).

### Variant calling and phylogenetic analyses.

Snippy v4.6.0 was used to generate a core genome alignment based on single nucleotide polymorphisms (SNPs) and insertions/deletions (indels) (42). The ‘snippy-clean_full_aln’ program was used to remove strange characters and the resultant alignment was analyzed for recombination with Gubbins v2.4.1 (43). The core alignment obtained after the removal of the polymorphic sites was used to generate a phylogenetic tree with IQtree v2.0.3 with 1000 bootstrap replicates and ascertainment bias correction (44). The best-fit substitution model detected was the transversion model with empirical base frequencies. The final phylogenetic tree was visualized with FigTree v1.4.4 (45). Final representation was performed using Phandango v1.3.0 that allowed the association with isolates’ metadata (46).

The resulting full and core alignments were also loaded in Unipro UGENE to generate new alignments using the multiple sequence comparison by log-expectation (MUSCLE) method (41, 47). These alignments were used to construct distance matrixes of both full and core alignments by Hamming dissimilarity and represent them by counts of dissimilarity.

Finally, to investigate if there was enough temporal signal in the data to perform phylogenetic molecular clock analysis, both TempEst v1.5.3 and BactDating v1.1 tools were implemented (48, 49).

### Virulence factors analysis.

Presence of virulence factors encoding genes were identified performing BLAST searches with Bandage (50). A database including *apxI*, *apxII*, *apxIII,* and *apxIV* genes was created, and serotype-specificity was studied. Gene encoding the ApxIV hemolysin was further analyzed using Unipro UGENE tools for specific sequence patterns (41, 51).

### Resistance profile, plasmid, and prophage identification.

From all annotated assemblies, resistance genes were identified using ABRicate v1.0.1 with the CARD database (52, 53). Identification of plasmids was performed with PLSDB v2021_06_23_v2 using the mash screen strategy with a maximum *P*-value of 0.1 and a minimum identity of 0.98 (54). Besides, the plasmidSPAdes algorithm v3.14.1 was used to perform plasmid assemblies from the raw reads of the 16 selected APP isolates of this study (55). BLAST search tool in the NCBI database was used to analyze resultant scaffolds for rapid sequence comparison (56). Potential active prophages were located and annotated with Prophage Hunter (accessed August 2021) and genomic islands were predicted with Alien Hunter (accessed June 2021) and visualized with Artemis v18.1.0 (57, 58).

### Isolation, identification, and antimicrobial susceptibility testing.

Clinical specimens isolated from Spanish farms were cultured aseptically, and identification of APP isolates was performed as previously described (6). MIC values were determined using the broth microdilution method (Sensititre, Trek diagnostic Systems Inc., East Grinstead, UK) and following the Clinical and Laboratory Standards Institute (CLSI) (59). The antimicrobials tested included amoxicillin, ceftiofur, doxycycline, enrofloxacin, florfenicol, marbofloxacin, oxytetracycline, sulfamethoxazole/trimethoprim, tiamulin, tilmicosin, tildipirosin, and tulathromycin (6).

Clinical breakpoints (CB) from CLSI were used to determine antimicrobial susceptibility (59, 60). However, CLSI CB for sulfamethoxazole/trimethoprim and *Pasteurellaceae* have not been set. Thus, the CLSI CB available for Streptococcus suis (0.5 μg/mL) and sulfamethoxazole/trimetoprim were used in this study. The CB for amoxicillin (0.5 μg/mL) was obtained from the literature and CLSI CB available for tetracycline (0.5 μg/mL) and enrofloxacin and porcine respiratory pathogens were extrapolated for doxycycline, oxytetracycline, and marbofloxacin, respectively (59–61).

### Pangenome construction.

Pangenome analyses were executed following the anvi’o v7 workflow for microbial pangenomics (62, 63). This workflow allowed the identification of gene clusters among the genomes under study. Prior to the pangenome construction, all genome FASTA files were reformatted and converted into an anvi’o contigs database with the ‘anvi-script-reformat-fasta’ and the ‘anvi-gen-contigs-database’ programs. The program ‘anvi-run-ncbi-cogs’ was used for gene annotation of the contigs.

For constructing the pangenome, the external genomes were first included in a new anvi’o genomes storage using the ‘anvi-gen-genomes-storage’ program. Following, the program ‘anvi-pan-genome’ run the pan-genomic analysis on all the stored genomes searching by amino acid sequence similarity with the NCBI’s blastp tool. Additional metadata was added with the ‘anvi-import-misc-data’ program and average nucleotide identity (ANI) was computed with the ‘anvi-compute-genome-similarity’ program using the pyANI tool (64).

Finally, the pangenome was visualized in the anvi’o interactive interface with the ‘anvi-display-pan’ program. For further analyses, the full pangenome was split into core and accessory bins, based on the gene clusters frequency across the genomes.

### Data availability.

The genome sequences of the 16 isolates have been deposited in NCBI under the BioProject accession number PRJNA781224. The accession numbers per isolate are listed in Table 1.
