# Supplementary material for: Revealing Genomic Insights of the Unexplored Porcine Pathogen Actinobacillus pleuropneumoniae Using Whole Genome Sequencing
Source: Microbiol Spectr. 2022 Jul 20;10(4):e01185-22. doi: 10.1128/spectrum.01185-22 (PMC9430968; doi:10.1128/spectrum.01185-22)
Supplement: Supplemental file 1 — Tables S1 to S3. Download spectrum.01185-22-s0001.pdf, PDF file, 1.3 MB [file spectrum.01185-22-s0001.pdf]

Supplemental Material. Table S1. Hamming distance matrix based on dissimilar counts of the core alignment generated by UGENE. Matrix colors represent different degrees of identity; green-colored cells show a high identity correlation between isolates, while red-colored cells represent low identity correlation.

	ASM1588_5b_X_2007	ASM16709_5_Argentina_2003	ASM1735746_5_China_2021	ASM1676271_1_China_2017	ASM343140_1_SouthKorea_2017	ASM17849_1_Argentina_2018	ASM329038_1_Argentina_2018	ASM17857_9_Netherlands_2010	**UG1871964S21_11_Spain_2018**	**UG1871964S6_11_Spain_2018**	**UG1970170_11_Spain_2019**	**UG1970316_11_Spain_2019**	ASM17861_11_Netherlands_2010	**UG1970269_11_Spain_2019**	**UG1970277_11_Spain_2019**	**UG1970987_11_Spain_2019**	**UG1971163_11_Spain_2019**	**UG1970667_11_Spain_2019**	**UG1971013_11_Spain_2019**	**UP1971676_11_Spain_2019**	**UG1970008_11_Spain_2019**	**UG1970106_11_Spain_2019**	ASM17859_10_X_2010	42650_D01_6_Denmark_1971	ASM17855_6_Australia_2010	ASM17929_6_Australia_2010	ASM17865_7_Hungary_2010	ASM29591_7_China_2012	ASM2040_7_Canada_2008	ASM81744_8_Brazil_2011	ASM81746_8_Brazil_2007	ASM81751_8_Brazil_2009	ASM81752_8_Brazil_2006	ASM81753_8_Brazil_2008	ASM81748_8_Brazil_2007	MIDG2331_8_UK_2015	57675_E01_2_Denmark_1973	ASM17851_2_X_2010	ASM17927_2_Australia_2010	ASM17863_12_Australia_2010	**UG1871511_13_Spain_2018**	**UP1971182_13_Spain_2019**	**UP1971607_13_Spain_2019**	ASM1668_3_China_2008	42650C01_4_USA_1980	ASM17853_4_Australia_2010
ASM1588_5b_X_2007	0																																													
ASM16709_5_Argentina_2003	183	0																																												
ASM1735746_5_China_2021	106	135	0																																											
ASM1676271_1_China_2017	2527	2556	2458	0																																										
ASM343140_1_SouthKorea_2017	2515	2544	2446	66	0																																									
ASM17849_1_Argentina_2018	2515	2544	2450	78	64	0																																								
ASM329038_1_Argentina_2018	2514	2543	2449	77	63	1	0																																							
ASM17857_9_Netherlands_2010	2522	2551	2453	106	92	92	91	0																																						
**UG1871964S21_11_Spain_2018**	2540	2569	2471	124	110	110	109	32	0																																					
**UG1871964S6_11_Spain_2018**	2540	2569	2471	124	110	110	109	32	0	0																																				
**UG1970170_11_Spain_2019**	2540	2569	2471	124	110	110	109	32	0	0	0																																			
**UG1970316_11_Spain_2019**	2540	2569	2471	124	110	110	109	32	0	0	0	0																																		
ASM17861_11_Netherlands_2010	2530	2559	2461	114	100	100	99	22	40	40	40	40	0																																	
**UG1970269_11_Spain_2019**	2539	2568	2470	123	109	109	108	31	49	49	49	49	17	0																																
**UG1970277_11_Spain_2019**	2541	2570	2472	125	111	111	110	33	51	51	51	51	19	4	0																															
**UG1970987_11_Spain_2019**	2538	2567	2469	122	108	108	107	30	48	48	48	48	16	1	3	0																														
**UG1971163_11_Spain_2019**	2539	2568	2470	123	109	109	108	31	49	49	49	49	17	2	4	1	0																													
**UG1970667_11_Spain_2019**	2544	2573	2475	128	114	114	113	36	54	54	54	54	22	15	17	14	15	0																												
**UG1971013_11_Spain_2019**	2539	2568	2470	123	109	109	108	31	49	49	49	49	17	20	22	19	20	25	0																											
**UP1971676_11_Spain_2019**	2538	2567	2469	122	108	108	107	30	48	48	48	48	16	19	21	18	19	24	1	0																										
**UG1970008_11_Spain_2019**	2514	2543	2445	140	124	126	125	88	106	106	106	106	96	105	107	104	105	110	105	104	0																									
**UG1970106_11_Spain_2019**	2514	2543	2445	140	124	126	125	88	106	106	106	106	96	105	107	104	105	110	105	104	0	0																								
ASM17859_10_X_2010	2412	2440	2343	2390	2376	2380	2379	2378	2398	2398	2398	2398	2388	2397	2399	2396	2397	2402	2397	2396	2376	2376	0																							
42650_D01_6_Denmark_1971	2220	2248	2148	2255	2241	2244	2243	2247	2267	2267	2267	2267	2257	2266	2268	2265	2266	2271	2266	2265	2245	2245	2153	0																						
ASM17855_6_Australia_2010	2219	2247	2147	2254	2240	2243	2242	2246	2266	2266	2266	2266	2256	2265	2267	2264	2265	2270	2265	2264	2244	2244	2152	1	0																					
ASM17929_6_Australia_2010	2220	2248	2148	2255	2241	2244	2243	2247	2267	2267	2267	2267	2257	2266	2268	2265	2266	2271	2266	2265	2245	2245	2153	4	3	0																				
ASM17865_7_Hungary_2010	2371	2400	2304	2280	2266	2270	2269	2270	2288	2288	2288	2288	2278	2287	2289	2286	2287	2292	2287	2286	2264	2264	2222	1893	1892	1893	0																			
ASM29591_7_China_2012	2370	2399	2303	2279	2265	2269	2268	2269	2287	2287	2287	2287	2277	2286	2288	2285	2286	2291	2286	2285	2263	2263	2221	1892	1891	1892	33	0																		
ASM2040_7_Canada_2008	2370	2399	2303	2279	2265	2269	2268	2269	2287	2287	2287	2287	2277	2286	2288	2285	2286	2291	2286	2285	2263	2263	2221	1892	1891	1892	39	38	0																	
ASM81744_8_Brazil_2011	2307	2337	2246	2372	2358	2361	2360	2362	2380	2380	2380	2380	2370	2379	2381	2378	2379	2383	2379	2378	2356	2356	2260	1827	1826	1827	2080	2079	2079	0																
ASM81746_8_Brazil_2007	2320	2350	2259	2385	2371	2374	2373	2375	2393	2393	2393	2393	2383	2392	2394	2391	2392	2396	2392	2391	2369	2369	2273	1840	1839	1840	2093	2092	2092	19	0															
ASM81751_8_Brazil_2009	2325	2355	2264	2390	2376	2379	2378	2380	2398	2398	2398	2398	2388	2397	2399	2396	2397	2401	2397	2396	2374	2374	2278	1845	1844	1845	2098	2097	2097	26	21	0														
ASM81752_8_Brazil_2006	2324	2354	2263	2391	2377	2380	2379	2381	2399	2399	2399	2399	2389	2398	2400	2397	2398	2402	2398	2397	2375	2375	2279	1844	1843	1844	2099	2098	2098	27	22	27	0													
ASM81753_8_Brazil_2008	2311	2341	2250	2376	2362	2365	2364	2366	2384	2384	2384	2384	2374	2383	2385	2382	2383	2387	2383	2382	2360	2360	2264	1831	1830	1831	2084	2083	2083	12	11	16	17	0												
ASM81748_8_Brazil_2007	2319	2349	2258	2384	2370	2373	2372	2374	2392	2392	2392	2392	2382	2391	2393	2390	2391	2395	2391	2390	2368	2368	2270	1839	1838	1839	2092	2091	2091	20	31	38	39	24	0											
MIDG2331_8_UK_2015	2306	2336	2245	2373	2359	2362	2361	2363	2381	2381	2381	2381	2371	2380	2382	2379	2380	2384	2380	2379	2357	2357	2261	1826	1825	1826	2081	2080	2080	11	22	27	26	13	21	0										
57675_E01_2_Denmark_1973	2354	2382	2286	2253	2239	2242	2241	2243	2261	2261	2261	2261	2251	2260	2262	2259	2260	2265	2260	2259	2237	2237	2329	1989	1988	1989	2156	2155	2155	2039	2052	2057	2056	2043	2051	2038	0									
ASM17851_2_X_2010	2355	2383	2287	2254	2240	2243	2242	2244	2262	2262	2262	2262	2252	2261	2263	2260	2261	2266	2261	2260	2238	2238	2330	1990	1989	1990	2157	2156	2156	2040	2053	2058	2057	2044	2052	2039	11	0								
ASM17927_2_Australia_2010	2351	2379	2283	2250	2236	2239	2238	2240	2258	2258	2258	2258	2248	2257	2259	2256	2257	2262	2257	2256	2234	2234	2326	1986	1985	1986	2153	2152	2152	2036	2049	2054	2053	2040	2048	2035	7	4	0							
ASM17863_12_Australia_2010	2408	2437	2341	2299	2285	2289	2288	2291	2309	2309	2309	2309	2299	2308	2310	2307	2308	2313	2308	2307	2281	2281	2199	2142	2141	2142	2220	2219	2219	2094	2107	2112	2111	2098	2106	2093	2008	2009	2005	0						
**UG1871511_13_Spain_2018**	2422	2451	2355	2278	2264	2267	2266	2272	2290	2290	2290	2290	2280	2289	2291	2288	2289	2294	2289	2288	2262	2262	2204	2023	2022	2023	2000	1999	1999	2116	2127	2132	2133	2118	2126	2115	2078	2079	2075	2077	0					
**UP1971182_13_Spain_2019**	2423	2452	2356	2279	2265	2268	2267	2273	2291	2291	2291	2291	2281	2290	2292	2289	2290	2295	2290	2289	2263	2263	2205	2024	2023	2024	2001	2000	2000	2117	2128	2133	2134	2119	2127	2116	2079	2080	2076	2078	1	0				
**UP1971607_13_Spain_2019**	2422	2451	2355	2278	2264	2267	2266	2272	2290	2290	2290	2290	2280	2289	2291	2288	2289	2294	2289	2288	2262	2262	2204	2023	2022	2023	2000	1999	1999	2116	2127	2132	2133	2118	2126	2115	2078	2079	2075	2077	0	1	0			
ASM1668_3_China_2008	2456	2485	2389	2238	2224	2227	2226	2228	2248	2248	2248	2248	2238	2247	2249	2246	2247	2252	2247	2246	2222	2222	2118	2016	2015	2016	2192	2191	2191	2012	2025	2030	2031	2016	2024	2013	2019	2020	2016	2097	2028	2029	2028	0		
42650C01_4_USA_1980	2298	2328	2236	2202	2188	2190	2189	2196	2214	2214	2214	2214	2206	2213	2215	2212	2213	2220	2215	2214	2190	2190	2298	2026	2025	2026	2175	2174	2176	1997	2010	2015	2014	2001	2009	1996	2133	2134	2130	2115	1991	1992	1991	1766	0	
ASM17853_4_Australia_2010	2298	2328	2236	2202	2188	2190	2189	2196	2214	2214	2214	2214	2206	2213	2215	2212	2213	2220	2215	2214	2190	2190	2298	2026	2025	2026	2175	2174	2176	1997	2010	2015	2014	2001	2009	1996	2133	2134	2130	2115	1991	1992	1991	1766	0	0

Supplemental Material. Table S2. Minimal Inhibitory Concentration (MIC) values for antimicrobial susceptibility testing of the 16 Spanish APP isolates. Antimicrobial susceptibility was determined using the CLSI clinical breakpoints. The antimicrobials tested included amoxicillin (AMO), ceftiofur (CEF), doxycycline (DOX), enrofloxacin (ENR), florfenicol (FLO), marbofloxacin (MAR), oxytetracycline (OXI), sulfamethoxazole/trimethoprim (SFM), tiamulin (TIA), tilmicosin (TIM), tildipirosin (TID) and tulathromycin (TUL).

Isolate ID	AMO	CEF	DOX	ENR	FLO	MAR	OXI	SFM	TIA	TIM	TID	TUL	Pattern
UP1971607	8	≤0.06	2	1	0.25	1	>8	≤0.12	16	16	4	32	i
UP1971182	16	≤0.06	2	4	0.25	4	>8	≤0.12	16	16	4	32	
UG1871511	8	≤0.06	2	1	0.25	1	>8	≤0.12	16	16	4	32	
UG1970106	0.25	≤0.06	2	≤0.03	0.25	≤0.03	>8	4	16	8	4	32	iii
UG1970008	0.25	≤0.06	2	≤0.03	0.25	≤0.03	>8	8	16	16	4	64	
UG1970316	0.25	≤0.06	0.5	≤0.03	0.25	≤0.03	0.5	≤0.12	16	8	8	32	ii
UG1871964S6	≤0.12	≤0.06	0.5	≤0.03	0.25	≤0.03	0.5	≤0.12	16	8	4	32	
UG1871964S21	≤0.12	≤0.06	0.5	≤0.03	0.25	≤0.03	0.5	≤0.12	16	8	4	32	
UG1970170	0.25	≤0.06	0.5	0.06	0.25	≤0.03	0.5	≤0.12	16	8	4	32	
UG1971013	≤0.12	≤0.06	0.5	0.06	0.25	0.06	0.5	≤0.12	16	8	8	32	
UP1971676	0.25	≤0.06	0.5	0.06	0.25	0.06	0.5	≤0.12	16	16	8	32	
UG1971163	≤0.06	≤0.06	0.25	≤0.03	0.12	≤0.03	0.25	≤0.06	4	4	2	16	
UG1970277	≤0.12	≤0.06	0.5	≤0.03	0.25	≤0.03	0.5	≤0.12	16	8	4	32	
UG1970269	≤0.12	≤0.06	0.5	≤0.03	0.25	0.06	0.5	≤0.12	16	8	8	64	
UG1970987	0.25	≤0.06	0.5	≤0.03	0.25	≤0.03	0.5	≤0.12	16	8	4	32	
UG1970667	≤0.12	≤0.06	0.5	≤0.03	0.25	≤0.03	0.5	≤0.12	8	16	8	64	

Supplemental Material. Table S3. List of the 21 active prophagues detected by the Prophague Hunter tool with the general characteristics obtained from the software.

Isolate ID	Sequence ID	Start	End	Length	Category	Score	Closest phage	Gene number
ASM1735746_5_China_2021	NZ_CP026009.1	661411	704179	42769	Active	0.98	Mannheimia phage vB_MhS_3927AP1	60
ASM1676271_1_China_2017	JAEKIR010000001.1	100243	142321	42079	Active	0.90	Mannheimia phage vB_MhS_3927AP1	56
UG1970170_11_Spain_2019	NODE_4_length_188174_cov_89.319782	47275	89375	42101	Active	0.91	Mannheimia phage vB_MhS_3927AP1	57
ASM17861_11_Netherlands_2010	ADOK01000012.1	46736	88836	42101	Active	0.91	Mannheimia phage vB_MhS_3927AP1	57
UG1970269_11_Spain_2019	NODE_4_length_188236_cov_69.291772	99277	141377	42101	Active	0.91	Mannheimia phage vB_MhS_3927AP1	57
UG1970277_11_Spain_2019	NODE_3_length_205851_cov_62.416573	46861	88961	42101	Active	0.91	Mannheimia phage vB_MhS_3927AP1	171
UG1970987_11_Spain_2019	NODE_4_length_188193_cov_73.521296	46861	88961	42101	Active	0.91	Mannheimia phage vB_MhS_3927AP1	57
UG1971163_11_Spain_2019	NODE_4_length_188237_cov_90.164111	99277	141377	42101	Active	0.91	Mannheimia phage vB_MhS_3927AP1	5
UG1971013_11_Spain_2019	NODE_4_length_188230_cov_77.856685	99271	141371	42101	Active	0.91	Mannheimia phage vB_MhS_3927AP1	57
UG1970008_11_Spain_2019	NODE_3_length_224407_cov_62.410273	82142	124226	42085	Active	0.92	Mannheimia phage vB_MhS_3927AP1	174
ASM17859_10_X_2010	ADOJ01000039.1	2203	23774	21572	Active	0.89	Streptococcus phage CHPC577	35
ASM17855_6_Australia_2010	ADOG01000011.1	116753	130944	14192	Active	0.96	Lactobacillus phage JCL1032	19
ASM17929_6_Australia_2010	ADXO01000035.1	75641	110052	34412	Active	0.99	Mannheimia phage vB_MhS_535AP2	41
ASM17865_7_Hungary_2010	ADOM01000010.1	146415	170148	23734	Active	0.84	Mannheimia phage vB_MhS_3927AP1	25
ASM29591_7_China_2012	ALYN01000033.1	24520	53790	29271	Active	0.98	Mannheimia phage vB_MhS_587AP2	41
ASM2040_7_Canada_2008	CP001091.1	893425	916746	23322	Active	0.87	Stenotrophomonas phage IME-SM1	32
ASM81752_8_Brazil_2006	JSVY01000008.1	3565	58497	54933	Active	0.84	Mannheimia phage vB_MhS_3927AP1	74
ASM81748_8_Brazil_2007	JSVZ01000007.1	47404	95702	48299	Active	0.97	Mannheimia phage vB_MhS_3927AP1	76
57675_E01_2_Denmark_1973	LR134515.1	484066	539365	55300	Active	0.90	Mannheimia phage vB_MhS_587AP2	68
42650_C01_4_USA_1980	LS483358.1	2078803	2105948	27146	Active	1.00	Mannheimia phage vB_MhM_3927AP2	42
ASM17853_4_Australia_2010	ADOF01000084.1	30162	45088	14927	Active	0.85	Mannheimia phage vB_MhM_3927AP2	15
